# Bacterial abundance and diversity in 64–74 Ma subseafloor igneous basement from the Louisville Seamount Chain

**DOI:** 10.1002/mlf2.12148

**Published:** 2024-12-24

**Authors:** Jason B. Sylvan, Benjamin J. Tully, Yuki Morono, Jeffrey C. Alt, Sharon L. Grim, Fumio Inagaki, Anthony A. P. Koppers, Katrina J. Edwards

**Affiliations:** ^1^ Department of Biological Sciences University of Southern California Los Angeles California USA; ^2^ Center for Dark Energy Biosphere Investigations University of Southern California Los Angeles California USA; ^3^ Kochi Institute for Core Sample Research, Japan Agency for Earth‐Marine Science and Technology (JAMSTEC) Nankoku, Kochi Japan; ^4^ Department of Earth and Environmental Sciences University of Michigan Ann Arbor Michigan USA; ^5^ Josephine Bay Paul Center for Comparative Molecular Biology and Evolution, Marine Biological Laboratory Woods Hole Massachusetts USA; ^6^ Advanced Institute for Marine Ecosystem Change (WPI‐AIMEC), JAMSTEC Yokohama Japan; ^7^ Department of Earth Sciences, Graduate School of Science Tohoku University Sendai Japan; ^8^ College of Earth, Ocean and Atmospheric Sciences Oregon State University Corvallis Oregon USA; ^9^ Present address: Department of Oceanography Texas A&M University College Station Texas USA; ^10^ Present address: Department of Marine Chemistry and Geochemistry Woods Hole Oceanographic Institution Woods Hole Massachusetts USA

## Abstract

The aquifer in the subseafloor igneous basement is a massive, continuous microbial substrate, yet sparingly little is known about life in this habitat. The work to date has focused largely on describing microbial diversity in the young basement (<10 Ma), where the basaltic crust is still porous and fluid flow through it is active. Here, we test the hypothesis that microbial life exists in subseafloor basement >65 Ma using samples collected from the Louisville Seamount Chain via seafloor drilling. Cell biomass was heterogeneous in nature, ranging from below detection to ~10^4^ cells cm^−3^. Bacterial 16S rRNA genes from core samples and enrichment incubations are dominated by lineages putatively carrying out nitrogen, sulfur, and metal redox processes and hydrocarbon oxidation. Taken together, the data indicate that microbial life is indeed present in subseafloor igneous basement >65 Ma, which significantly expands the range of the subseafloor biosphere where microbial life is known to exist.

The entire volume of the ocean cycles through subseafloor basaltic crust every 10^5^–10^6^ years^1^, and the volume of the habitable zone in the marine basement is equivalent to the entire volume of the marine water column^2^. As such, the basaltic rock that comprises much of the upper oceanic crust represents a massive, globally distributed potential microbial habitat. Despite the potential importance and magnitude of the microbial biosphere in subseafloor basalts, knowledge about this biosphere remains extremely limited, partially due to the difficulty of sampling its environment^3^. To date, only a few studies have focused on microbial processes in subseafloor basalts, and primarily targeting young basalts <10 Ma^4^, ^5^, ^6^, ^7^, ^8^. These pioneering work indicates that microbial communities in young subseafloor basement are heterogeneous.

Efforts to understand microbial life in older settings are even more scarce and have been largely hampered by low biomass recovery and drilling contamination^3^. As a global average, oceanic hydrothermal circulation is predicted to cease at 65 Ma due to crustal sealing^9^, so interest has generally been higher in the younger crust with potentially high rates of fluid flow. However, there is indeed evidence for fluid flow in the older crust^10^, suggesting that a subseafloor biosphere may exist there. In fact, recent evidence from the basement underlying the South Pacific Gyre revealed locally high cell densities at grain boundaries, potentially up to 10^10^ cells cm^−3^ in samples from 33.5 to 104 Ma basalt^11^, but this tantalizing evidence was restricted to two samples.

It is known that seamounts provide conduits of fluid flow into and out of basement^12^, and that this is true even in basement >65 Ma^13^, indicating that seamounts located on older parts of the ocean crust may be home to subsurface microbial populations carried there by entrained seawater or resident since sometime after eruptive activity created the seamount. To test the hypothesis that subseafloor igneous rocks in seamounts >65 Ma host microbial populations, and to expand our understanding of life in one of the most expansive yet understudied biomes on earth, we collected samples cored during seafloor drilling of extinct seamounts along the Louisville Seamount Chain during Integrated Ocean Drilling Program (IODP) Expedition 330^14^. Drilled rock cores were collected from 29 to 491 meters below seafloor (mbsf) from Holes U1372A (74.2 Ma), U1373A (69.5 Ma), U1374A (67.4–71.1 Ma), and U1376A (64.1 Ma) (Table S1 and Figure 1A), all consisting of basement created at the Louisville hotspot in the south Pacific Ocean and described as alternating units of sediment or volcaniclastic breccia and erupted basalt^14^. Here, we report results from those samples for cell enumeration, measurement of organic carbon content, δ^13^C stable isotope analysis, and 16S rRNA amplicon analysis of rock core samples and enrichment cultures.

**Figure 1:**
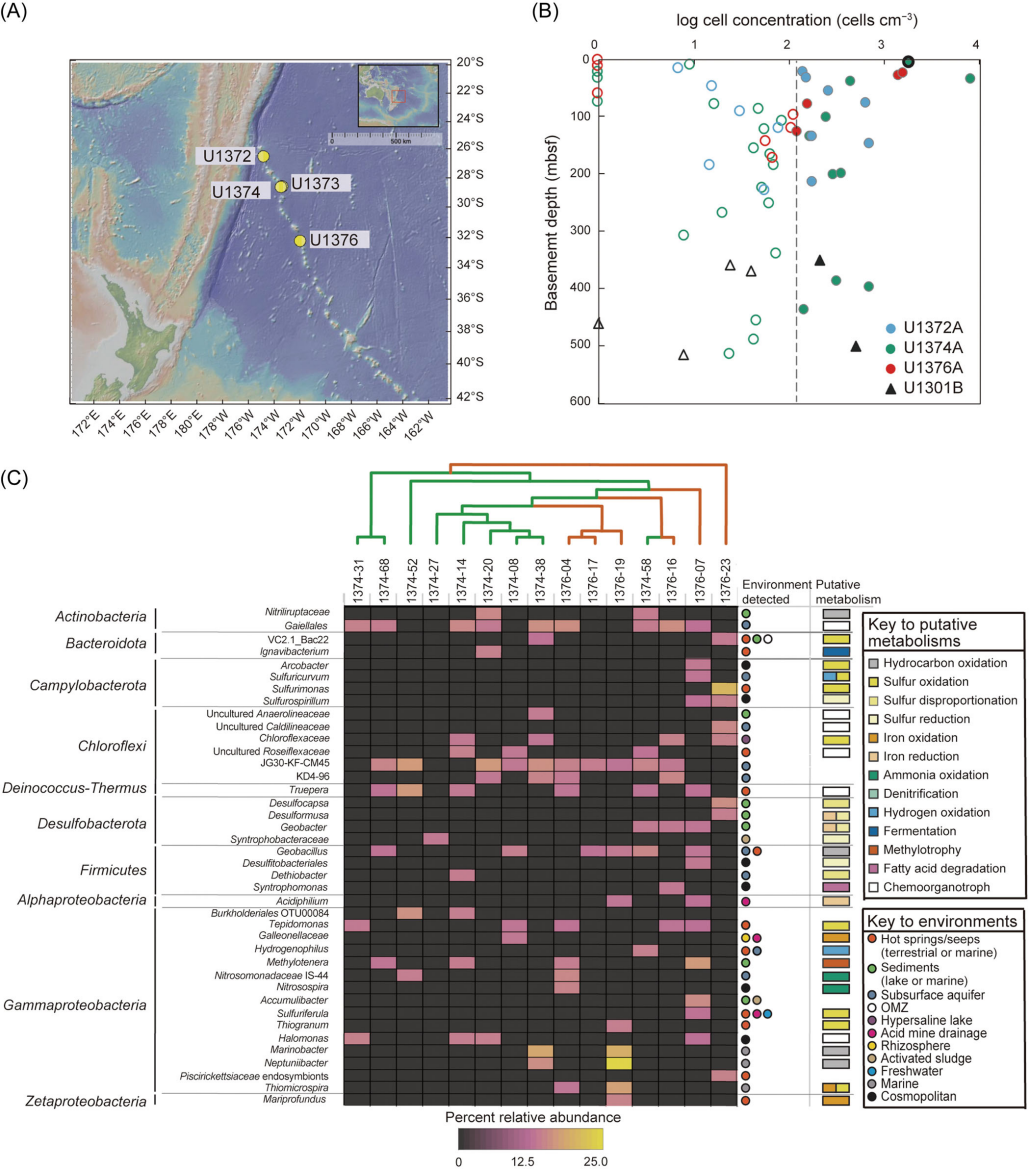
Microbial abundance and bacterial diversity in the subsurface Louisville Seamount ecosystem. (A) Map of Sites drilled and cored during Integrated Ocean Drilling Program (IODP) Expedition 330 from which samples were analyzed for this project. The image was made with GeoMapApp (www.geomapapp.org)/CC BY/CC BY (Ryan et al.)^15^. (B) Subseafloor cell counts from IODP Expeditions to the Louisville Seamounts (Expedition 330, U1372A, U1374A, and U1376A) and the Juan de Fuca Ridge (JdFR) flank (Expedition 301, U1301B). The dotted line indicates the limit of quantification, values to the left are considered below quantification. The sample from Site U1374A circled in black represents a sediment sample overlying the basement at that site. (C) Heatmap of OTUs most likely endemic to the subsurface Louisville Seamounts, organized by phylum (at left) and lowest classification possible (each row) and sample (columns). A Jaccard dissimilarity cladogram was at the top, with samples from Hole U1374A in green and Hole U1376A in red. The environments from which each OTU was typically detected are listed at right, next to the putative metabolism for those OTUs where significant cultured representatives have been studied.

We quantified cell density in 53 samples from the Louisville Seamounts: 13 from Canopus Guyot (Site U1372A), 29 from Rigil Guyot (Site U1374A), and 11 from Burton Guyot (Site U1376A). We also analyzed 6 samples from Site U1301B on the Juan de Fuca Ridge (JdFR) flank for context. Prokaryotic cell abundance in our samples ranges from below detection (no cells detected on the filter) to 8.3 × 10^4^ cells cm^−3^ with a minimum quantifiable limit of 1.20 × 10^2^ cells cm^−3^ (Figure 1B). Cell concentrations from the young JdFR flank are similar to those from the Louisville Seamounts, and the single sediment sample falls within the expected range for samples from the South Pacific Gyre^16^, in which Site U1374A is located. There is no apparent relationship between lithology or depth and biomass.

The cell concentrations detected here are similar in magnitude to the deep subseafloor basement in the JdFR, evidenced by whole‐round core samples analyzed here as well as observatory fluid samples from Site U1362, which range from 2.6 × 10^3^ to 2.6 × 10^4^ cells cm^−3^ ^5^. Similar concentration ranges were also detected in the cool, oxic ridge flank environment at the North Pond study site, where cell concentrations in whole‐round core samples range from below detection to 6 × 10^4^ cells cm^−3^ ^17^, and fluid samples from borehole observatories range 1× 10^4^ to 2 × 10^4^ cells cm^−3^ ^8^, as well as in lower oceanic crust from 10 to 781 mbsf at the 12 Ma Atlantis Bank^6^. Cell densities in shallow Atlantis Massif, ~1.5 Ma, are quite low, ranging from below detection to 4 × 10^3^ cells cm^−3^ ^4^. Cell concentrations in the whole‐rock samples from North Pond, Atlantis Bank, and Atlantis Massif are also very heterogeneous. Data from the Louisville Seamount Chain and JdFR presented here, coupled with the data from these other basement sites, add to the growing consensus that heterogeneous abundance with depth is likely a defining characteristic of subseafloor basement microbial communities. This indicates that there are fundamental differences between controls on subsurface life in marine basement versus sediments. We hypothesize that this is driven by fluid flow pathways in the basement, which are heterogeneous, whereas biomass distribution in sediments is largely constrained by conditions such as concentrations of oxygen and labile organic matter in overlying waters^16^. Therefore, predictions of global biomass in the subseafloor aquifer should account for fracture density and porosity and permeability structure in the basement.

Weight % total C, total organic carbon (TOC), and δ^13^C for total C and TOC were measured from a subset of the samples analyzed for biomass that had enough material (*n* = 8). Weight % TOC ranges from 0.006 to 0.154 (Table S2), which is generally lower than subseafloor basalts collected from Hole U1301B^18^. No relationship is evident between microbial cell counts and weight % TOC, suggesting that microbial biomass makes up a small percentage of TOC in the Louisville Seamounts basement. δ^13^C‐TOC values are −17.8‰ to −23.7‰, which is slightly higher than for subseafloor basalts from JdFR, −21.6‰ to −34.6‰, which were interpreted to reflect the presence of microbial organic carbon in the rocks^18^. The δ^13^C‐TOC values for the Louisville seamounts are generally similar to that for dissolved organic carbon (DOC) in seawater (δ^13^C = −20‰ to −22‰), and the simplest explanation is that seawater is the source, with no fractionation occurring in the subsurface. An alternative hypothesis is that the TOC is a product of microbial activity in the rocks with seawater carbonate or DOC as the source, which is supported by indications of recycling of organic matter at Site U1473 on Atlantis Bank^6^. The slightly more negative δ^13^C‐TOC values (to −23.7‰) could be the result of microbial activity, similar to that at JdFR^18^. Some samples from borehole U1301B on the JdFR are heterogeneous, with some similar to seawater DOC, while others are in the −27‰ to −34‰ range, indicating that microbial effects in basement rocks are variable, depending on fluid flow pathways in the basement, as well as heterogeneity of alteration effects.

We used amplicon sequencing of the V4V6 region of the bacterial 16S rRNA gene^19^ to examine bacterial diversity in 15 samples from Holes U1374A (9 samples, 40–491 mbsf) and U1376A (6 samples, 29–174 mbsf; Table S1). Almost no archaeal 16S rRNA genes were detected via qPCR for all of these samples except for 1376‐23 (see discussion in SOM for further details). Similar low archaeal abundance was detected in subsurface basalt samples from North Pond^20^ and gabbroic samples from Atlantis Bank^6^. This is not the case at the JdFR sites^5^, ^18^, which are warm (~65°C) and anoxic. Altogether, these findings support the trend that Archaea have a low abundance on cold silicate rocks.

Mean bacterial communities sampled from Hole U1376A were more diverse than those from Hole U1374A (Table S3 and Figure S1), but this was only statistically significant for richness (*t*‐test, *p* < 0.023). The most abundant phyla represented in the subseafloor Louisville Seamount environment are the *Actinobacteriota*, *Bacteroidota*, *Firmicutes*, and *Proteobacteria* (Figure S2). Within these phyla, the classes *Actinobacteria*, *Bacteroidia*, *Clostridia*, and *Alpha*‐ and *Gammaproteobacteria* are the most abundant.

For those top five most abundant classes, one to six orders per lineage represented the majority of diversity present (Figure S3). Among these, *Corynebacterales*, *Bacillales*, *Clostridiales*, *Rhizobiales*, *Burkholderiales*, and *Xanthomonadales* were the most abundant. *Burkholderiales* were also abundant community members in subseafloor basalts underlying North Pond^20^. *Actinobacteria* and *Firmicutes* were found to be more abundant at U1374A and U1376A than at the North Pond holes.

Analysis of community richness reveals that microbial communities in samples from Holes U1374A and U1376A are most similar to other samples from the same Hole (Figure 1C). More specifically, two clusters are apparent: microbial communities from samples 1374‐08, 1374‐14, 1374‐20, 1374‐27, and 1374‐38, all from Hole U1374A, grouped together, while communities from samples 1376‐04, 1376‐17, and 1376‐19, from Hole U1376A, formed a separate group. This indicates that location and/or seamount has some impact on community structure in this environment. Importantly, there were no apparent patterns between microbial communities and lithology, indicating that the individual seamount, and therefore Site location, or perhaps age of substrate (since age varies by seamount), may have a more important impact in this case than lithology.

We focused on the analysis of genera and families detected that may be indicative of a subsurface lifestyle based on where cultured representatives or environmental sequences have been predominately detected (Figure 1C). Of these, there was a predominance of putative metabolisms related to S, N, and Fe redox chemistry and hydrocarbon oxidation. The detection of metabolisms related to S and Fe redox chemistry is notable because basalts are composed of ~1% S and 10% Fe; therefore, these sources of metabolic energy are inherent in the substrate. In particular, *Zetaproteobacteria* related to *Mariprofundis ferooxidans*, an obligate Fe‐oxidizer^21^, were detected in the Louisville Seamount Chain subsurface. *Zetaproteobacteria* were also detected in the North Pond observatories, where metagenomic data indicates they are fixing carbon and oxidizing Fe^8^. Other putative iron oxidizers in the Louisville Seamount Chain subsurface include *Thiomicrospira* ^22^ and *Gallionaceae*. Putative iron reducers include *Geobacter*, *Desulfuromusa*, and *Acidiphilium*. Putative sulfur oxidizers and reducers were distributed within the *Bacteroidota*, *Campylobacterota*, *Desulfobacterota*, *Firmicutes*, and *Gammaproteobacteria* (Figure 1C).

Several genera putatively capable of hydrocarbon oxidation were also detected, including *Nitriliruptaceae*, *Geobacillus*, *Thauera*, *Marinobacter*, and *Neptuniibacter*. The latter two, in particular, have been noted for their role in hydrocarbon oxidation in the marine environment^23^, ^24^, ^25^. Subsurface communities detected in gabbro below Atlantis Massif and Atlantis Bank included abundant hydrocarbon degraders^6^, ^7^, indicating that this may be a common subsurface phenotype.

To determine if microorganisms from subsurface seamount environments could be stimulated to grow, we initiated enrichment experiments with core samples from Holes U1372A, U1373A, U1374A, and U1376A during Expedition 330 and extracted DNA from those experiments 171–1281 days later (Table S4). We used growth media targeting chemoorganotrophs, iron reducers, and sulfur oxidizers, likely metabolisms based on the presence of S and Fe in basalts. Analysis of V4V6 16S rRNA amplicons from the 35 enrichment incubations yielded additional insight beyond the amplicon analysis of the cored samples (Figure S4). In particular, several taxa were recovered from both the enrichments and the core samples. These include the VC2.1_Bac22 clade of *Bacteroidia*, *Sulfurimonas* (*Campylobacterota*), *Methylotenera, Thiomicrospira*, and *Halomonas* (*Gammaproteobacteria*) and *Mariprofundus* (*Zetaproteobacteria*; Figure S5). Additionally, the taxa *Sphingobacteriaceae* (*Bacteroidia*), the *Ktedonobacteria* taxa B12‐WMSP1, and the genus *Gemmatimonas* in the class *Gemmatimonadetes* were found exclusively in enrichments with added Fe(III), indicating a possible role in Fe‐cycling. Cultivated members of *Thiomicrospira*, *Zetaproteobacteria*, and *Sulfurimonas* are autotrophic^22^, ^26^ and therefore the amplicons detected here putatively represent members of the base of the subsurface food web in the Louisville ecosystem.

We show here that there is a subseafloor crustal biome in regions of seafloor comprised of basement >65 Ma that is unique from that found in younger settings previously studied. This is important because it reveals that the subseafloor crustal biome cannot be treated as a monolithic entity as we endeavor to understand global patterns. It also indicates that considerations of global subsurface biomass must include marine basement of all ages. Further, the biomass values measured here, in addition to previous work at North Pond^8^, ^17^, ^20^, Atlantis Massif^4^, and Atlantis Bank^6^, indicate a subseafloor biome with lower cell densities than overlying sediments. However, subseafloor basement is a spatially vast biosphere, including hotspots of biomass at mid‐ocean ridges and upper oceanic crust^11^, so its population size, geographical distribution, metabolic activity, and ecological significance remain largely unknown. Future work will help build a global database of subseafloor microbiomes in this least‐explored crustal biosphere.

## AUTHOR CONTRIBUTIONS

**Jason B. Sylvan**: Conceptualization (equal); data curation (lead); formal analysis (lead); funding acquisition (equal); investigation (lead); methodology (equal); project administration (lead); resources (equal); supervision (equal); visualization (equal); writing—original draft (equal); writing—review and editing (equal). **Benjamin J. Tully**: Data curation (equal); formal analysis (equal); investigation (equal); methodology (equal); visualization (equal); writing—original draft (equal); writing—review and editing (equal). **Yuki Morono**: Formal analysis (equal); investigation (equal); methodology (equal); resources (equal); supervision (equal); writing—review and editing (equal). **Jeffrey C. Alt**: Data curation (equal); formal analysis (equal); resources (equal); writing—review and editing (equal). **Sharon L. Grim**: Data curation (supporting); methodology (equal); writing—review and editing (equal). **Fumio Inagaki**: Conceptualization (equal); funding acquisition (equal); methodology (equal); resources (equal); writing—review and editing (equal). **Anthony A. P. Koppers**: Conceptualization (equal); funding acquisition (equal); resources (equal); supervision (equal); writing—review and editing (equal). **Katrina J. Edwards**: Conceptualization (equal); funding acquisition (equal); methodology (supporting); resources (equal); supervision (equal).

## ETHICS STATEMENT

This study did not involve any human participants or animal subjects.

## CONFLICT OF INTERESTS

The authors declare no conflict of interests.

## Supporting information

Supporting information.

Supporting information.

## Data Availability

All DNA sequence data are available in the NCBI Sequence Read Archive under Bioproject PRJNA271884, accession numbers SRX834467–SRX834482 (core samples), Bioproject PRJNA1033664, accession numbers SAMN38041014–SAMN38041051 (enrichment samples), and Genbank accession numbers OR751581–OR751585 (representative clones from sample 1372‐18‐HSO).
