# Supplementary material for: Bacterial abundance and diversity in 64–74 Ma subseafloor igneous basement from the Louisville Seamount Chain
Source: mLife. 2024 Dec 24;3(4):578–83. doi: 10.1002/mlf2.12148 (PMC11685831; doi:10.1002/mlf2.12148)
Supplement: Supplementary file 1 — Supporting information. [file MLF2-3-578-s001.docx]

***Supporting Information for:***

**Bacterial abundance and diversity in 64-74 Ma subseafloor igneous basement from the Louisville Seamount Chain**

Jason B. Sylvan^1,9,*^, Benjamin J. Tully^1,2^, Yuki Morono^3^, Jeffrey C Alt^4^, Sharon L. Grim^5^, Fumio Inagaki^6,7^, Anthony A.P. Koppers^8^ & Katrina J. Edwards^1,2,#^

^1^Department of Biological Sciences, University of Southern California, Los Angeles, CA 90089, USA

^2^Center for Dark Energy Biosphere Investigations, University of Southern California, Los Angeles, CA 90089, USA

^3^Kochi Institute for Core Sample Research, Japan Agency for Earth-Marine Science and Technology (JAMSTEC), Nankoku, Kochi 783-8502, Japan

^4^Department of Earth and Environmental Sciences, University of Michigan, Ann Arbor, MI 48109, USA

^5^Josephine Bay Paul Center for Comparative Molecular Biology and Evolution, Marine Biological Laboratory, Woods Hole, MA 02543, USA

^6^Advanced Institute for Marine Ecosystem Change (WPI-AIMEC), JAMSTEC, Yokohama 236-00001, Japan.

^7^Department of Earth Sciences, Graduate School of Science, Tohoku University, Sendai 980-8574, Japan

^8^College of Earth, Ocean and Atmospheric Sciences, Oregon State University, Corvallis, Oregon 97331, USA

^9^*current address:* Department of Oceanography, Texas A&M University, College Station, TX 77845, USA

^#^Deceased

*Correspondence: jasonsylvan@tamu.edu

**Materials and Methods**

*Core Handling and Sampling*

All samples were collected using rotary core barrel drilling during IODP Expedition 330, 13 December 2010 – 11 February 2011 (Figure 1). Detailed sampling methods are published elsewhere^1^ and summarized here. Whole-round cores were selected in the core splitting room and collected from the core liner onto pre-combusted (450˚C for 2 hours) aluminum foil. Sections were specifically chosen that showed some sign of alteration or a fluid flow conduit because these are likely locations for microbial life. Microbiology samples ranged 5–14 cm long. Prior work has shown that the interior of rock cores is generally free from contamination^2^, therefore, efforts were taken to sample only the interior of the cores. The intact whole round was washed 3X with artificial seawater in a fresh ziplock bag for each rinse before subsampling to avoid contamination from drilling fluids. Next, the rock was split with a flame-sterilized sterile chisel and sampled for the interiors of the cores. Samples were placed in 5 mL, autoclaved centrifuge tubes and immediately put at -80˚C for later analysis.

*Contamination testing onboard the JOIDES Resolution*

As part of the drilling process, huge amounts of surface seawater mixed with a proprietary chemical mix are injected into boreholes; this fluid, known as drilling fluid or drilling mud, is the major source of contamination of microorganisms in cores collected during coring. To check for contamination, the microbial composition of drilling fluid was assessed (Sample 1373-DF). 1373-DF was collected directly from the injection pipe on deck into sterile bottles with screw caps and then frozen at -80°C until processing. Once thawed, microorganisms present in fluids were collected by filtration using a vacuum pump onto a 0.2 µm pore polycarbonate filter. DNA was extracted as described below immediately following filtration.

Bags of yellow to green fluorescent microspheres (Fluoresbrite carboxylate microspheres; Polysciences Inc. 15700) with a diameter of 0.52 (±0.01) μm were used as a particulate tracer that mimics microbial cells on the *JOIDES Resolution* ^3^. These microspheres were counted in core samples for an indication of contamination on 2-3 cores per site. The concentration of microspheres was set at 10^10^ spheres/ml^3^. Concentrations of fluorescent microspheres in core samples were quantified using a Zeiss Axiophot epifluorescence microscope outfitted with a mercury lamp (HBO 100W), a blue filter set, and a 100Å~ Plan-NEOFLUAR oil-immersion objective. Non-fluorescent immersion oil was used for all observations. Aliquots (100μL) of the crushed rock were suspended into 10 ml of filtered 1xPBS solution and filtered onto black, 25-mm-diameter polycarbonate filters (0.2-μm pore size) in a filtration tower. The microspheres on the filter were then counted using the epifluorescence microscope. Microsphere abundance on the filters was determined by averaging the total number seen in at least 20 randomly selected fields of view and by looking at outer, inner and center portions of the core.

*Biomass enumeration*

A cell extraction and enumeration method originally developed for quantifying microbial biomass in subseafloor sediments^4^ and recently adapted for samples from ocean crust^5,6^ was used. Briefly, frozen samples were powderized in a tungsten carbide mortar and pestle that was previously decontaminated with RNAse Away, and then fixed in sterile filtered 2% formaldehyde at a volume:volume ratio of 1:5. One ml of the fixed sample slurry was used in the quantification procedure, which was followed as in Morono et al. (2013) except that 40 cycles of sonication were used instead of 20 to liberate cells attached to the powdered rock. Cells were enumerated on filters stained with 1/40 SYBR Green 1 in TE buffer by counting either 800–900 fields of view if fewer than 40 cells total were detected, or at least 40–50 cells in fewer fields when possible. The limit of quantification was defined as 3X the standard deviation of the mean of the negative control counts. Two negative controls were processed and analyzed for every ten experimental samples.

*DNA extraction and sequencing from cores*

Core samples were extracted as previously^7^ using a CTAB phenol/chloroform protocol with 1% CTAB. Starting material for amplicon sequencing samples was ~4 cm^3^ of rock chips. With the exception of sample 1376-23, all other samples analyzed by amplicon analysis were purified using synchronous coefficient of drag alteration (SCODA)^8^, as implemented with disposable cartridges and the Aurora System (Boreal Genomics, Mountain View, CA). A SCODA negative control was processed by running ultrapure deionized water in an Aurora cartridge and sequencing the output.

*Enrichment experiments*

Enrichment experiments were started during Expedition 330 (see Table S3 for details). For each, ~1 cm^3^ was added to a serum vial with 5 ml media. The media used targeted heterotrophs (1% Marine Broth and 10% Marine Broth), heterotrophic sulfur oxidizers (HSO), autotrophic iron reducers (AIR) or heterotrophic iron reducers (HIR) using recipes published previously^9^. All enrichments were incubated at 4˚C until processed for DNA extractions. Five ml of enrichment culture was filtered onto a 0.2 μm pore-size polycarbonate filter and frozen at -80˚C until community DNA was extracted using either the same method as used for the core samples, MoBio PowerWater DNA Isolation Kit, or MP Biomedical FastDNA Kit for Soil, as indicated in Table S4. One DNA extraction blank (no sample added to DNA extraction procedure) was also processed and used for contamination control. Samples were sent to Research and Testing Lab for PCR of the V4V6 region of 16S rRNA using the same primers as the core samples and then sequencing using 454 pyrosequencing with a target of 3000 reads per sample. One sample, 1372-18-HSO, was processed using the bacterial primers 27F (5'-GAG TTT GAT CCT GGC TCA G-3') and 519R (5'-GTA TTA CCG CGG CTG CTG G-3') for PCR followed by cloning and sequencing of the PCR products. The PCR product was run on an agarose gel, cut out, and extracted using QIAquick Gel Extraction Kit (Qiagen, Valencia, CA, USA) according to the manufacturer’s instructions. Fragments were cloned into the pCR 4 TOPO vector using the TOPO TA Cloning Kit (Invitrogen, Grand Island, NY, USA) and transformants plated on LB+100 mg mL^-1^ ampicillin according to the manufacturer’s instructions. Colonies were randomly selected and grown in liquid culture followed by sequencing at Beckman Coulter Genomics (Danvers, MA). Only one enrichment sample was analyzed using clone libraries because analysis via 454 sequencing became possible for this project after that initial sample was analyzed and 454 sequencing provides more data, so we moved forward using that methodology for the remainder of the project. However, results from the clone library were still valuable, so included here.

The 454 amplicon datasets were processed in mothur^10^ using the same protocol as for the core samples. Clones were assessed via BLAST to determine identity at the genus level; only five unique sequences were recovered.

*Archaea specific quantitative Polymerase Chain Reaction (qPCR)*

qPCR was used to estimate relative abundance of Archaea as described previously ^7^ using the primers 806f (5’-ATT AGA TAC CCS BGT AGT-3’ ^11^) and 922r (5’-YCC GGC GTT GAN TCC AAT T-3’ ^12^). The thermal program employed was: 10 minutes at 95˚C followed by 45 cycles of 30 sec. at 95˚C, 30 sec. at 55˚C and 25 sec. at 72˚C. Melt curves for all qPCR products were checked to ensure a single PCR product was generated. qPCR reactions were run in triplicate and the limit of detection was 54 gene copies per reaction, the mean of qPCR negative control reactions with water added instead of sample.

*16S rRNA Amplicon Analysis*

V4V6 amplicons were analyzed using the software package mothur ^10^ in two rounds, adapting elements of a protocol used to determine contamination in deep subsurface sediment samples ^13^. OTUs have been found to yield similar ecological results to amplicon sequence variants ^14^, including in deep subsurface biosphere environments ^15^, and were used here. For the first round, OTUs were generated at the 95% cutoff level. All OTUs were generated using the pre.cluster option in Mothur, which uses modified single-linkage ^16^ to account for sequencing errors. Four mismatches were allowed per cluster, equivalent to one mismatch per 100 bp ^17^ and the average neighbor method was used for OTU clustering. To control from contamination, OTUs detected in either the negative control samples (1373-DF or AuroraBlank) at an average relative abundance of ≥10X than they were detected in the average of the 15 experimental samples were removed. Additionally, taxa were removed that were previously detected in sequencing kits as contaminants (^18^, Table S5). The contaminant subtracted amplicon libraries from the 15 experimental samples were then run through the same analysis pipeline in mothur starting from the raw sequences, this time using 97% similarity cutoff to generate OTUs for comparison of OTU abundance per sample and 95% for comparison of community similarity between samples, as has been done previously ^7,19^. The same process was used for the enrichment samples, except that a blank DNA extraction sample (LouiBlank) was used as the negative control.

**Supplemental Results**

*Discussion of qPCR results*

qPCR was performed on samples after they were cleaned up with the Boreal Genomics Aurora Purification System, as described in the Materials and Methods section. Bacterial 16S rRNA was quantified in triplicate as described previously^20^ using primers 338f (5’-ACT CCT ACG GGA GGC AGC AG-3') and 518r (5'-ATT ACC GCG GCT GCT GG-3'. qPCR for Archaea was carried out as described in Materials and Methods. Because there was obvious background contamination in the Aurora cartridges, we decided the data for bacterial biomass is unreliable. However, because archaeal 16S rRNA was below detection, we believe this reflects the environment accurately because the contamination should result in either similar or increased 16S rRNA gene copies. Dilution below the detection limit is also possible, but it should be noted that other similar environments also have low abundance of Archaea^6,21^. Therefore, the lack of archaeal amplification is reported as part of our analysis since it indicates a true lack of archaeal 16S rRNA gene copies, but the bacterial quantification is not reported as it will clearly be biased.

*Putative contaminant removal*

To control for potential contamination of our samples by drilling fluid or during DNA extraction and cleanup, we first generated OTUs at the 95% similarity level and subtracted OTUs detected in negative controls at abundances 10X higher than experimental samples. The 10X threshold was used to allow for the possibility that some OTUs may show up in the controls but be much more abundant in the samples, indicating they are likely true members of the community in that sample. This approach avoids overly conservative discarding of any sequence detected in the controls but is still strict enough to minimize the likelihood of contaminant sequences remaining after QC. After removing contaminants detected at the 95% OTU level, we reanalyzed the sequences that passed QC by constructing OTUs at 97% similarity cutoff. Following this quality control step, there were 385–5873 bacterial V4V6 amplicons per sample (Table S1). The percent of amplicons remaining following blank subtraction ranged ~2–37% with a mean value of 16%.

*Zetaproteobacteria in the enrichments*

Genera and families detected in this study and in other subsurface settings, including hot springs, cold seeps, sediments and subsurface aquifers, are referenced here^6,25-49^. In total, five Zetaproteobacterial OTUs^22^ were detected in the enrichment experiments (Table S5). This notably includes ZetaOTU9, considered a subsurface clade^23^, and most closely related to the recently described *Ghiorsea bivora*, which can oxidize both Fe and H^24^. ZetaOTU11, represented by *M. ferrooxidans*, was also detected, as was ZetaOTU58, recently recovered from mild steel incubated in sediments^25^.

**Supplementary Information References**

1. Expedition 330 Scientific Party. Louisville Seamount Trail: implications for geodynamic mantle flow models and the geochemical evolution of primary hotspots. IODP Preliminary Report, 330. 2011:doi:10.2204/iodp.pr.330.011.

2. Lever M, Alperin M, Engelen B, Inagaki F, Nakagawa S, Steinsbu B, et al. Trends in Basalt and Sediment Core Contamination During IODP Expedition 301. Geomicrobiol. J. 2006;23(7):517-30.

3. Smith DC, Spivack AJ, Fisk MR, Haveman SA, Staudigel H, Party tLSS. Methods for quantifying potential microbial contamination during deep ocean coring. ODP Technical Note. 2000;28.

4. Morono Y, Terada T, Kallmeyer J, Inagaki F. An improved cell separation technique for marine subsurface sediments: applications for high-throughput analysis using flow cytometry and cell sorting. Environ Microbiol. 2013;15(10):2841-9.

5. Früh-Green GL, Orcutt BN, Rouméjon S, Lilley MD, Morono Y, Cotterill C, et al. Magmatism, serpentinization and life: Insights through drilling the Atlantis Massif (IODP Expedition 357). Lithos. 2018;323:137-55.

6. Wee SY, Edgcomb VP, Beaudoin D, Yvon-Lewis S, Sylvan JB. Microbial Abundance and Diversity in Subsurface Lower Oceanic Crust at Atlantis Bank, Southwest Indian Ridge. Appl Environ. Microbiol. 2021;87(22):e0151921.

7. Sylvan JB, Sia TY, Haddad AG, Briscoe LJ, Toner BM, Girguis PR, Edwards KJ. Low temperature geomicrobiology follows host rock composition along a geochemical gradient in lau basin. Front Microbiol. 2013;4:61.

8. Engel K, Pinnell L, Cheng J, Charles TC, Neufeld JD. Nonlinear electrophoresis for purification of soil DNA for metagenomics. J. Microbiol. Meth. 2012;88(1):35-40.

9. Expedition 330 Scientists. Methods. In: Koppers AAP, Yamazaki T, Geldmacher J, the Expedition 330 Scientists, editors. Proceedings of the Integrated Ocean Drilling Program. 330. Tokyo: IODP Management International, Inc.; 2012.

10. Schloss PD, Westcott SL, Ryabin T, Hall JR, Hartmann M, Hollister EB, et al. Introducing mothur: Open-Source, Platform-Independent, Community-Supported Software for Describing and Comparing Microbial Communities. Appl. Environ. Microbiol. 2009;75(23):7537-41.

11. Takai K, Horikoshi K. Rapid detection and quantification of members of the archaeal community by quantitative PCR using fluorogenic probes. Appl. Environ. Microbiol. 2000;66(11):5066-72.

12. DeLong EF. Archaea in coastal marine environments. Proc. Natl. Acad. Sci. U.S.A. 1992;89:5685-9.

13. Inagaki F, Hinrichs KU, Kubo Y, Bowles MW, Heuer VB, Hong WL, et al. Exploring deep microbial life in coal-bearing sediment down to ~2.5 km below the ocean floor. Science. 2015;349(6246):420-4.

14. Glassman SI, Martiny JBH. Broadscale Ecological Patterns Are Robust to Use of Exact Sequence Variants versus Operational Taxonomic Units. mSphere. 2018;3(4).

15. Kerrigan Z, D'Hondt S. Patterns of Relative Bacterial Richness and Community Composition in Seawater and Marine Sediment Are Robust for Both Operational Taxonomic Units and Amplicon Sequence Variants. Front. Microbiol. 2022;13:796758.

16. Huse SM, Welch DM, Morrison HG, Sogin ML. Ironing out the wrinkles in the rare biosphere through improved OTU clustering. Environ. Microbiol. 2010;12(7):1889-98.

17. Schloss PD, Gevers D, Westcott SL. Reducing the effects of PCR amplification and sequencing artifacts on 16S rRNA-based studies. PLoS One. 2011;6(12):e27310.

18. Salter SJ, Cox MJ, Turek EM, Calus ST, Cookson WO, Moffat MF, et al. Reagent and laboratory contamination can critically impact sequence-based microbiome analyses. BMC Biol. 2014;12:87.

19. Toner BM, Lesniewski RA, Marlow JJ, Briscoe LJ, Santelli CM, Bach W, et al. Mineralogy Drives Bacterial Biogeography of Hydrothermally Inactive Seafloor Sulfide Deposits. Geomicrobiol. J. 2013;30(4):313-26.

20. Einen J, Thorseth IH, Ovreas L. Enumeration of Archaea and Bacteria in seafloor basalt using real-time quantitative PCR and fluorescence microscopy. FEMS Microbiol Lett. 2008;282(2):182-7.

21. Jorgensen SL, Zhao R. Microbial inventory of deeply buried oceanic crust from a young ridge flank. Front. Microbiol. 2016;7:820.

22. McAllister SM, Moore RM, Chan CS. ZetaHunter, a Reproducible Taxonomic Classification Tool for Tracking the Ecology of the Zetaproteobacteria and Other Poorly Resolved Taxa. Microbiol. Resourc. Announc. 2018;7(7): e00932-18.

23. McAllister SM, Davis RE, McBeth JM, Tebo BM, Emerson D, Moyer CL. Biodiversity and emerging biogeography of the neutrophilic iron-oxidizing Zetaproteobacteria. Appl. Environ. Microbiol. 2011;77(15):5445-57.

24. Mori JF, Scott JJ, Hager KW, Moyer CL, Kusel K, Emerson D. Physiological and ecological implications of an iron- or hydrogen-oxidizing member of the Zetaproteobacteria, *Ghiorsea bivora*, gen. nov., sp. nov. ISME J. 2017;11(11):2624-36.

25. Barco RA, Hoffman CL, Ramirez GA, Toner BM, Edwards KJ, Sylvan JB. In-situ incubation of iron-sulfur mineral reveals a diverse chemolithoautotrophic community and a new biogeochemical role for *Thiomicrospira*. Environ. Microbiol. 2017;19(3):1322-37.

26. Albuquerque L, França L, Rainey FA, Schumann P, Nobre MF, da Costa MS. Gaiella occulta gen. nov., sp. nov., a novel representative of a deep branching phylogenetic lineage within the class Actinobacteria and proposal of Gaiellaceae fam. nov. and Gaiellales ord. nov. Syst. Appl. Microbiol. 2011;34(8):595-9.

27. Albuquerque L, Simoes C, Nobre MF, Pino NM, Battista JR, Silva MT, et al. Truepera radiovictrix gen. nov., sp. nov., a new radiation resistant species and the proposal of Trueperaceae fam. nov. FEMS Microbiol. Lett. 2005;247(2):161-9.

28. Breusing C, Castel J, Yang Y, Broquet T, Sun J, Jollivet D, et al. Global 16S rRNA diversity of provannid snail endosymbionts from Indo-Pacific deep-sea hydrothermal vents. Environ. Microbiol. Rep. 2022;14(2):299-307.

29. Campbell BJ, Engel AS, Porter ML, Takai K. The versatile epsilon-proteobacteria: key players in sulphidic habitats. Nat. Rev. Microbiol. 2006;4(6):458-68.

30. Doyle SM, Whitaker EA, De Pascuale V, Wade TL, Knap AH, Santschi PH, et al. Rapid Formation of Microbe-Oil Aggregates and Changes in Community Composition in Coastal Surface Water Following Exposure to Oil and the Dispersant Corexit. Front. Microbiol. 2018;9:689.

31. Finster K, Liesack W, Thamdrup B. Elemental sulfur and thiosulfate disproportionation by Desulfocapsa sulfoexigens sp. nov., a new anaerobic bacterium isolated from marine surface sediment. Appl. Environ. Microbiol.. 1998;64(1):119-25.

32. Gonzalez-Pimentel JL, Martin-Pozas T, Jurado V, Miller AZ, Caldeira AT, Fernandez-Lorenzo O, et al. Prokaryotic communities from a lava tube cave in La Palma Island (Spain) are involved in the biogeochemical cycle of major elements. PeerJ. 2021;9:e11386.

33. Holmes DE, Nevin KP, Lovley DR. Comparison of 16S rRNA, nifD, recA, gyrB, rpoB and fusA genes within the family Geobacteraceae fam. nov. Int. J. Syst. Evol. Microbiol. 2004;54(5):1591-9.

34. Iino T, Mori K, Uchino Y, Nakagawa T, Harayama S, Suzuki K. Ignavibacterium album gen. nov., sp. nov., a moderately thermophilic anaerobic bacterium isolated from microbial mats at a terrestrial hot spring and proposal of Ignavibacteria classis nov., for a novel lineage at the periphery of green sulfur bacteria. Int. J. Syst. Evol. Microbiol. 2010;60(Pt 6):1376-82.

35. Jakus N, Blackwell N, Straub D, Kappler A, Kleindienst S. Presence of Fe(II) and nitrate shapes aquifer-originating communities leading to an autotrophic enrichment dominated by an Fe(II)-oxidizing Gallionellaceae sp. FEMS Microbiol. Ecol. 2021;97(11).

36. Kaye JZ, Sylvan JB, Edwards KJ, Baross JA. Halomonas and Marinobacter ecotypes from hydrothermal vent, subseafloor and deep-sea environments. FEMS Microbiol. Ecol. 2011;75(1):123-33.

37. Kimura H, Asada R, Masta A, Naganuma T. Distribution of microorganisms in the subsurface of the manus basin hydrothermal vent field in Papua New Guinea. Appl. Environ. Microbiol. 2003;69(1):644-8.

38. Kojima H, Kanda M, Umezawa K, Fukui M. Sulfurimicrobium lacus gen. nov., sp. nov., a sulfur oxidizer isolated from lake water, and review of the family Sulfuricellaceae to show that it is not a later synonym of Gallionellaceae. Arch. Microbiol. 2021;203(1):317-23.

39. Madigan MT, Schaaf NAV, Sattley WM. The Chlorobiaceae, Chloroflexaceae, and Heliobacteriaceae. In: Hallenbeck PC, editor. Modern Topics in the Phototrophic Prokaryotes: Environmental and Applied Aspects. Cham: Springer International Publishing; 2017. p. 139-61.

40. Monteverde DR, Sylvan JB, Suffridge C, Baronas JJ, Fichot E, Fuhrman J, et al. Distribution of extracellular flavins in a coastal marine basin and their relationship to redox gradients and microbial community members. Environ. Sci. Technol. 2018;52(21):12265-74.

41. Mori K, Suzuki KI, Yamaguchi K, Urabe T, Hanada S. Thiogranum longum gen. nov., sp. nov., an obligately chemolithoautotrophic, sulfur-oxidizing bacterium of the family Ectothiorhodospiraceae isolated from a deep-sea hydrothermal field, and an emended description of the genus Thiohalomonas. Int. J. Syst. Evol. Microbiol. 2015;65(Pt 1):235-41.

42. Neubeck A, Sun L, Müller B, Ivarsson M, Hosgörmez H, Özcan D, et al. Microbial community structure in a serpentine-hosted abiotic gas seepage at the Chimaera Ophiolite, Turkey. Appl. Environ. Microbiol. 2017;83(12):e03430-16.

43. Sorokin DY, Chernyh N. ‘Candidatus Desulfonatronobulbus propionicus’: a first haloalkaliphilic member of the order Syntrophobacterales from soda lakes. Extremophiles. 2016;20(6):895-901.

44. Sorokin DY, van Pelt S, Tourova TP, Evtushenko LI. Nitriliruptor alkaliphilus gen. nov., sp. nov., a deep-lineage haloalkaliphilic actinobacterium from soda lakes capable of growth on aliphatic nitriles, and proposal of Nitriliruptoraceae fam. nov. and Nitriliruptorales ord. nov. Int. J. Syst. Evol. Microbiol. 2009;59(2):248-53.

45. Suzuki S, Ishii S, Wu A, Cheung A, Tenney A, Wanger G, et al. Microbial diversity in The Cedars, an ultrabasic, ultrareducing, and low salinity serpentinizing ecosystem. Proc. Natl. Acad. Sci. U.S.A. 2013;110(38):15336-41.

46. Sylvan JB, Pyenson BC, Rouxel O, German CR, Edwards KJ. Time series analysis of two hydrothermal plumes at 9 ̊50'N East Pacific Rise reveals distinct, heterogeneous bacterial populations. Geobiology. 2012;10:178-92.

47. Tiago I, Verissimo A. Microbial and functional diversity of a subterrestrial high pH groundwater associated to serpentinization. Environ. Microbiol. 2013;15(6):1687-706.

48. Watson SJ, Needoba JA, Peterson TD. Widespread detection of Candidatus Accumulibacter phosphatis, a polyphosphate-accumulating organism, in sediments of the Columbia River estuary. Environ. Microbiol. 2019;21(4):1369-82.

49. Wilkins LGE, Ettinger CL, Jospin G, Eisen JA. Metagenome-assembled genomes provide new insight into the microbial diversity of two thermal pools in Kamchatka, Russia. Sci. Rep. 2019;9(1):3059.

**Supplementary Online Tables**

Table S1 – Sample descriptions and cell biomass. Short sample names are the site number followed the core number (e.g. 1374-07 for sample U1374A-7R1).

| Sample | Short Name | Sample Collection Date | Depth Below Seafloor (mbsf) | Lithology | | Cell Biomass (cells cm^-3^) | | # Amplicons  post-QC (raw dataset) | |
| --- | --- | --- | --- | --- | --- | --- | --- | --- | --- |
| *Hole U1374A, Rigil Guyot, 28.596 S, 173.381 W, water depth 1545 m* | | | | |  | |  | |  |
| U1374A-08-R-1 | 1374-08 | 07-Jan-2011 | 39.6 | Volcanic sandstone | | 457 | | 1872 (19,550) | |
| U1374A-14-R-2 | 1374-14 | 07-Jan-2011 | 75 | Sedimentary breccia | | bd | | 3141 (97850 | |
| U1374A-20-R-1 | 1374-20 | 08-Jan-2011 | 102.9 | Aphyric basalt breccia | | 247 | | 1442 (15,019) | |
| U1374A-27-R-1 | 1374-27 | 10-Jan-2011 | 135.8 | Olivine-plagioclase-augite phyric basalt breccia | | 168 | | 3067 (23,012) | |
| U1374A-31-R-3 | 1374-31 | 11-Jan-2011 | 167.2 | Olivine phyric basalt breccia | | bql | | 385 (23,866) | |
| U1374A-38-R-2 | 1374-38 | 12-Jan-2011 | 203.6 | Olivine phyric basalt breccia | | 292 | | 2302 (17,388) | |
| U1374A-52-R-4 | 1374-52 | 14-Jan-2011 | 340.8 | Plagioclase-augite-olivine phyric basalt breccia | | bql | | 2280 (18,870) | |
| U1374A-58-R-6 | 1374-58 | 15-Jan-2011 | 400.2 | Plagioclase phyric basalt breccia | | 716 | | 4346 (22,606) | |
| U1374A-68-R-2 | 1374-68 | 17-Jan-2011 | 491.2 | Aphyric basalt breccia | | bql | | 2149 (23,691) | |
| *Hole U1376A, Burton Guyot, 32.217 S, 171.881 W, water depth 1550 m* | | | | |  | |  | |  |
| U1376A-04-R-1 | 1376-04 | 28-Jan-2011 | 29.4 | Boundstone (carbonate) | | 1443 | | 2514 (17,866) | |
| U1376A-07-R-3 | 1376-07 | 29-Jan-2011 | 60.7 | Olivine-augite phyric basalt breccia | | bd | | 5873 (15,719) | |
| U1376A-16-R-5 | 1376-16 | 01-Feb-2011 | 120.8 | Olivine phyric basalt breccia | | bql | | 2590 (20,280) | |
| U1376A-17-R-2 | 1376-17 | 01-Feb-2011 | 126.7 | Olivine phyric basalt breccia | | 124 | | 2445 (16,727) | |
| U1376A-19-R-1 | 1376-19 | 01-Feb-2011 | 144.6 | Olivine phyric basalt breccia | | bql | | 5419 (23,542) | |
| U1376A-23-R-1 | 1376-23 | 02-Feb-2011 | 174.2 | Olivine phyric basalt breccia | | bql | | 2235 (20,391) | |
| *Negative Control samples* | |  |  |  | |  | |  | |
| U1373A-Drill Fluid | 1373-DF | 02-Jan-2011 | 0 (collected on deck) | Drilling mud collected from pipe on deck | | nd | | 25,991 (nd) | |
| Aurora Blank | AuroraBlank | 01 March 2013 | -- | Negative control blank for cleanup cartridge | | nd | | 12,407 (nd) | |

bd – below detection (no cells detected), bql – below quantification limit (<100 cells cm^-3^), nd – not determined

Table S2 - Summary of δ^13^C-Total C, Total C content, δ^13^C-TOC and TOC content

| Sample | Depth Below Seafloor (mbsf) | Cell Biomass (cells cm^-3^) | Total C  δ^13^C ‰VPDB | Total C  (wt %) | TOC  δ^13^C ‰VPDB | TOC  (wt %) |
| --- | --- | --- | --- | --- | --- | --- |
| U1374A-07-R-1 | 35.4 | 8334 | 2.02 | 0.377 | -20.36  (n=2) | 0.007  (n=2) |
| U1374A-20-R-1 | 102.9 | 247 | 2.67 | 3.231 | -20.54  (n=2) | 0.013  (n=2) |
| U1374A-27-R-1 | 135.8 | 168 | 2.74 | 3.465 | -21.67 | 0.010 |
| U1374A-38-R-2 | 203.6 | 292 | 2.85 | 1.535 | -20.11 | 0.008 |
| U1374A-58-R-6 | 400.2 | 716 | -6.99  (n=2) | 0.142  (n=2) | -17.83 | 0.014 |
| U1376A-04-R-1 | 29.4 | 1443 | 3.77 | 11.776 | -23.68 | 0.154 |
| U1376A-17-R-2 | 126.7 | 124 | -1.59 | 0.767  (n=2) | -23.048  (n=2) | 0.006  (n=2) |
| U1376A-19-R-1 | 144.6 | bql | 0.46 | 1.703 |  |  |

Table S3 - Diversity statistics for bacterial pyrotags

| Short Sample Name | # OTUs | Chao 1 | ACE |
| --- | --- | --- | --- |
| 1374-08 | 175 | 307 | 236 |
| 1374-14 | 232 | 317 | 281 |
| 1374-20 | 180 | 271 | 224 |
| 1374-27 | 149 | 317 | 236 |
| 1374-31 | 163 | 913 | 1786 |
| 1374-38 | 218 | 323 | 289 |
| 1374-52 | 100 | 408 | 698 |
| 1374-58 | 271 | 715 | 859 |
| 1374-68 | 138 | 610 | 685 |
| 1376-04 | 275 | 803 | 743 |
| 1376-07 | 325 | 919 | 1605 |
| 1376-16 | 260 | 837 | 875 |
| 1376-17 | 257 | 635 | 817 |
| 1376-19 | 173 | 433 | 615 |
| 1376-23 | 209 | 510 | 592 |

Table S4 - Sample data for enrichment incubations.

| Sample | Depth (mbsf) | Date Sampled on Exp330 | Transfer Date | Days Incubated | DNA Extraction Method | | # Pyrotags (post-subtraction) | |  |
| --- | --- | --- | --- | --- | --- | --- | --- | --- | --- |
| U1373A-2R1-1MB | 10.8 | 1-Jan-11 | 17-Aug-11 | 228 | | MoBio PowerWater | | 2386 (2386) | |
| U1374A-58R6-1MB | 400.2 | 15-Jan-11 | 5-Jul-11 | 171 | | MoBio PowerWater | | 1899 (1175) | |
| U1374A-6R3-10MB | 33.4 | 7-Jan-11 | 7-Nov-13 | 1035 | | MP Biomedical Fast Soil | | 3280 (3280) | |
| U1374A-7R1-10MB | 35.4 | 7-Jan-11 | 25-Jan-13 | 749 | | MP Biomedical Fast Soil | | 1397 (1389) | |
| U1374A-7R1-10MB | 35.4 | 7-Jan-11 | 7-Nov-13 | 1035 | | MP Biomedical Fast Soil | | 1462 (1462) | |
| U1372A-4R3-HSO | 16.9 | 22-Dec-10 | 17-Aug-11 | 238 | | MoBio PowerWater | | 1420 (1418) | |
| U1372A-18R2-HSO | 135.3 | 25-Dec-10 | 17-Aug-11 | 235 | | MoBio PowerWater | | NA | |
| U1373A-2R1-HSO | 10.8 | 1-Jan-11 | 17-Aug-11 | 228 | | MoBio PowerWater | | 1293 (1293) | |
| U1374A-6R3-HSO | 33.4 | 7-Jan-11 | 12-Sep-11 | 248 | | CTAB Phenol/Chloro | | 6392 (3995) | |
| U1376A-17R2-HSO | 127.8 | 1-Feb-11 | 25-Aug-11 | 205 | | CTAB Phenol/Chloro | | 1688 (1688) | |
| U1372A-4R3-HIR-1 | 16.9 | 22-Dec-10 | 23-Jun-14 | 1279 | | MP Biomedical Fast Soil | | 6258 (166) | |
| U1372A-4R3-HIR-2 | 16.9 | 22-Dec-10 | 25-Jun-14 | 1281 | | MP Biomedical Fast Soil | | 4620 (4) | |
| U1372A-8R5-HIR | 48.0 | 23-Dec-10 | 25-Jun-14 | 1280 | | MP Biomedical Fast Soil | | 3467 (93) | |
| U1372A-18R2-HIR | 135.3 | 25-Dec-10 | 23-Jun-14 | 1276 | | MP Biomedical Fast Soil | | 2023 (10) | |
| U1372A-29R1-HIR-1 | 186.2 | 27-Dec-10 | 25-Jun-14 | 1276 | | MP Biomedical Fast Soil | | 2674 (4) | |
| U1373A-2R1-HIR-1 | 10.8 | 1-Jan-11 | 25-Jun-14 | 1271 | | MP Biomedical Fast Soil | | 3116 (7) | |
| U1374A-4R1-HIR-1 | 21.5 | 6-Jan-11 | 24-Jun-14 | 1265 | | MP Biomedical Fast Soil | | 2568 (3) | |
| U1374A-4R1-HIR-2 | 21.5 | 6-Jan-11 | 25-Jun-14 | 1266 | | MP Biomedical Fast Soil | | 3136 (93) | |
| U1374A-6R3-HIR-1 | 33.4 | 7-Jan-11 | 23-Jun-14 | 1263 | | MP Biomedical Fast Soil | | 747 (25) | |
| U1374A-6R3-HIR-2 | 33.4 | 7-Jan-11 | 30-Jun-14 | 1270 | | MP Biomedical Fast Soil | | 364 (6) | |
| U1374A-7R1-HIR-1 | 35.4 | 7-Jan-11 | 24-Jun-14 | 1264 | | MP Biomedical Fast Soil | | 6231 (15) | |
| U1374A-7R1-HIR-2 | 35.4 | 7-Jan-11 | 25-Jun-14 | 1265 | | MP Biomedical Fast Soil | | 4177 (39) | |
| U1374A-20R1-HIR | 102.9 | 8-Jan-11 | 25-Jun-14 | 1264 | | MP Biomedical Fast Soil | | 5554 (18) | |
| U1374A-30R3-HIR | 156.8 | 10-Jan-11 | 25-Jun-14 | 1262 | | MP Biomedical Fast Soil | | 4938 (2) | |
| U1376A-16R5-HIR | 120.8 | 31-Jan-11 | 23-Jun-14 | 1239 | | MP Biomedical Fast Soil | | 8512 (5) | |
| U1376A-17R2-HIR | 127.8 | 1-Feb-11 | 23-Jun-14 | 1238 | | MP Biomedical Fast Soil | | 5379 (44) | |
| U1376A-23R1-HIR | 174.2 | 2-Feb-11 | 25-Jun-14 | 1239 | | MP Biomedical Fast Soil | | 6701 (8) | |
| U1372A-4R3-AIR-1 | 16.9 | 22-Dec-10 | 30-Jun-14 | 1286 | | MP Biomedical Fast Soil | | 1205 (21) | |
| U1372A-4R3-AIR-2 | 16.9 | 22-Dec-10 | 30-Jun-14 | 1286 | | MP Biomedical Fast Soil | | 4454 (4143) | |
| U1372A-29R1-AIR-2 | 186.2 | 27-Dec-10 | 30-Jun-14 | 1281 | | MP Biomedical Fast Soil | | 1484 (21) | |
| U1374A-4R1-AIR-2 | 21.5 | 6-Jan-11 | 2-Jul-14 | 1273 | | MP Biomedical Fast Soil | | 2003 (141) | |
| U1374A-7R1-AIR-1 | 35.4 | 7-Jan-11 | 2-Jul-14 | 1272 | | MP Biomedical Fast Soil | | 571 (8) | |
| U1376A-16R5-AIR | 120.8 | 31-Jan-11 | 7-Jul-14 | 1253 | | MP Biomedical Fast Soil | | 5281 (3) | |
| U1374A-20R1-AIR | 102.9 | 8-Jan-11 | 30-Jun-14 | 1269 | | MP Biomedical Fast Soil | | 2349 (63) | |
| U1374A-30R3-AIR-1 | 156.8 | 10-Jan-11 | 30-Jun-14 | 1267 | | MP Biomedical Fast Soil | | 3153 (15) | |
| Extraction Blank | NA | NA | NA | NA | | MP Biomedical Fast Soil | | 73 (0) | |

Table S5 - Assignments of OTUs defined as *Zetaproteobacteria* to Zetaproteobacterial OTUs (ZetaOTUs), as defined by Zetahunter.

| OTU name | Sample | ZetaOTU |
| --- | --- | --- |
| LouiC2_E7 | U1372-18R2-HSO | ZetaOtu9 |
| LouiC2_H10 | U1372-18R2-HSO | ZetaOtu9 |
| Lou10_II8GYES02HQNI9_Otu270 | U1374-58R6-1MB | ZetaOtu9 |
| Lou11_IJDV81G03GGH4L_Otu029 | U1374-6R3-HSO | ZetaOtu9 |
| Lou11_IJDV81G03FXDQE_Otu754 | U1374-6R3-HSO | ZetaOtu9 |
| Lou11_IJDV81G03GAEBC_Otu907 | U1374-6R3-HSO | ZetaOtu9 |
| Otu00110 | U1376-19R1 | ZetaOtu11 |
| Lou10_II8GYES02JGD7D_Otu134 | U1374-58R6-1MB | ZetaOtu58 |
| Lou10_II8GYES02GW1OC_Otu908 | U1374-58R6-1MB | ZetaOtu58 |
| Lou10_II8GYES02GU5GW_Otu906 | U1374-58R6-1MB | ZetaOtu58 |
| Lou10_II8GYES02HBNCU_Otu955 | U1374-58R6-1MB | ZetaOtu58 |
| LouiC2_D6 | U1373-4R3-HSO | ZetaOtu58 |
| Lou11_IJDV81G03G1PT5_Otu011 | U1374-6R3-HSO | ZetaOtu58 |
| Lou11_IJDV81G03HBVVZ_Otu910 | U1374-6R3-HSO | ZetaOtu58 |
| Lou11_IJDV81G03GZGB1_Otu422 | U1374-6R3-HSO | NewZetaOtu1 |
| Lou11_IJDV81G03F7296_Otu390 | U1374-6R3-HSO | NewZetaOtu2 |

Table S6 - Cell count data used to make Figure 1B.

| Site | Core | | Type | | Section | | depth (mbsf) | | cell count (cells/cm^-3^) | |  |
| --- | --- | --- | --- | --- | --- | --- | --- | --- | --- | --- | --- |
| 1372 | 4 | | R | | 4 | | 16.9 | | 7.0 | |  |
| 1372 | 5 | | R | | 4 | | 23.5 | | 143.4 | |  |
| 1372 | 7 | | R | | 1 | | 32.9 | | 152.2 | |  |
| 1372 | 8 | | R | | 5 | | 48 | | 15.5 | |  |
| 1372 | 9 | | R | | 4 | | 55.3 | | 267.5 | |  |
| 1372 | 11 | | R | | 5 | | 77.4 | | 654.6 | |  |
| 1372 | 13 | | R | | 2 | | 91.5 | | 30.5 | |  |
| 1372 | 16 | | R | | 2 | | 121.8 | | 78.8 | |  |
| 1372 | 18 | | R | | 2 | | 135.3 | | 174.2 | |  |
| 1372 | 21 | | R | | 2 | | 147.6 | | 702.6 | |  |
| 1372 | 29 | | R | | 1 | | 186.2 | | 15.0 | |  |
| 1372 | 34 | | R | | 1 | | 214.2 | | 179.7 | |  |
| 1372 | 38 | | R | | 1 | | 229.7 | | 55.3 | |  |
| 1374 | 1 | | R | | 4 | | 5.95 | | 1837.3 | |  |
| 1374 | 2 | | R | | 1 | | 10.59 | | 9.1 | |  |
| 1374 | 4 | | R | | 1 | | 21.5 | | 0.1 | |  |
| 1374 | 6 | | R | | 3 | | 33.4 | | 0.1 | |  |
| 1374 | 7 | | R | | 1 | | 35.4 | | 8337.8 | |  |
| 1374 | 8 | | R | | 1 | | 39.6 | | 456.9 | |  |
| 1374 | 14 | | R | | 2 | | 75 | | 0.1 | |  |
| 1374 | 15 | | R | | 2 | | 79.7 | | 16.7 | |  |
| 1374 | 17 | | R | | 1 | | 87.6 | | 48.3 | |  |
| 1374 | 20 | | R | | 1 | | 102.9 | | 246.6 | |  |
| 1374 | 21 | | R | | 2 | | 109 | | 86.4 | |  |
| 1374 | 24 | | R | | 3 | | 123.6 | | 56.3 | |  |
| 1374 | 27 | | R | | 1 | | 135.8 | | 167.7 | |  |
| 1374 | 30 | | R | | 3 | | 156.8 | | 43.4 | |  |
| 1374 | 31 | | R | | 3 | | 167.2 | | 64.8 | |  |
| 1374 | 34 | | R | | 3 | | 185.3 | | 69.4 | |  |
| 1374 | 37 | | R | | 4 | | 200.4 | | 364.2 | |  |
| 1374 | 38 | | R | | 2 | | 203.6 | | 291.6 | |  |
| 1374 | 40 | | R | | 5 | | 225.6 | | 52.3 | |  |
| 1374 | 43 | | R | | 2 | | 251.9 | | 62.2 | |  |
| 1374 | 45 | | R | | 2 | | 270.2 | | 20.1 | |  |
| 1374 | 49 | | R | | 3 | | 457.3 | | 45.5 | |  |
| 1374 | 52 | | R | | 4 | | 340.8 | | 74.9 | |  |
| 1374 | 57 | | R | | 4 | | 388.8 | | 325.8 | |  |
| 1374 | 58 | | R | | 6 | | 400.2 | | 715.9 | |  |
| 1374 | 62 | | R | | 6 | | 439.3 | | 147.8 | |  |
| 1374 | 64 | | R | | 5 | | 310.2 | | 8.0 | |  |
| 1374 | 68 | | R | | 2 | | 491.2 | | 43.3 | |  |
| 1374 | 71 | | R | | 3 | | 516.4 | | 23.7 | |  |
| 1376 | 1 | | R | | 2 | | 1.95 | | 0.1 | |  |
| 1376 | 2 | | R | | 3 | | 12.3 | | 0.1 | |  |
| 1376 | 3 | | R | | 5 | | 24.9 | | 1613.3 | |  |
| 1376 | 4 | | R | | 1 | | 28.9 | | 1443.0 | |  |
| 1376 | 7 | | R | | 3 | | 60.7 | | 0.1 | |  |
| 1376 | 9 | | R | | 2 | | 78.1 | | 159.3 | |  |
| 1376 | 12 | | R | | 2 | | 98.9 | | 112.0 | |  |
| 1376 | 16 | | R | | 5 | | 120.8 | | 106.3 | |  |
| 1376 | 17 | | R | | 2 | | 127.8 | | 124.2 | |  |
| 1376 | 19 | | R | | 1 | | 144.6 | | 56.7 | |  |
| 1376 | 23 | | R | | 1 | | 174.2 | | 67.2 | |  |
| 1301B | 1 | | R | | 1 | | 352 | | 207.9 | |  |
| 1301B | 2 | | R | | 2 | | 359.6 | | 24.2 | |  |
| 1301B | 4 | | R | | 4 | | 371.2 | | 40.1 | |  |
| 1301B | 17 | | R | | 1 | | 462.1 | | 0.1 | |  |
| 1301B | 23 | | R | | 2 | | 501.6 | | 507.4 | |  |
| 1301B | 26 | | R | | 1 | | 516.3 | | 7.8 | |  |
| *Blanks* |  | |  | |  | |  | |  | |  |
| 25apr_neg1 | |  | |  | |  | |  | | 98.1 | |
| 25apr_neg2 | |  | |  | |  | |  | | 47.5 | |
| 19apr_b1 | |  | |  | |  | |  | | 146.8 | |
| 16apr_neg1 | |  | |  | |  | |  | | 54.2 | |
| 29jan2014_neg1 | |  | |  | |  | |  | | 15.2 | |
| 29jan2014_neg2 | |  | |  | |  | |  | | 0.0 | |
| 03feb2014-neg1 | |  | |  | |  | |  | | 37.9 | |
| 03feb2014-neg2 | |  | |  | |  | |  | | 47.3 | |
| 07feb2014-neg2 | |  | |  | |  | |  | | 72.1 | |
| 11feb2014-neg2 | |  | |  | |  | |  | | 31.2 | |
| 11feb2014-neg1 | |  | |  | |  | |  | | 29.1 | |
| 07feb2014-neg1 | |  | |  | |  | |  | | 83.4 | |

**Supplementary Online Figures**

Figure S1 - Box and whisker plots for number of bacterial OTUs, defined at the 97% similarity cutoff, Chao1 and ACE for samples from Holes U1374A and U1376A.

Figure S2 – Bacterial classes detected through amplicon sequencing of the V4V6 region of 16S rRNA. "Others" represents taxa not present at >1% in any sample. Numbers of amplicons per sample are indicated in Table 1.

Figure S3 - Relative abundance of bacterial families representing ≥1% of the total dataset. Order and family names are on the left, Phylum and class (for Proteobacteria) are on the right. Color code matches class in Figure S2.

Fig. S4 - Bacterial families detected in enrichment incubations through pyrotag sequencing of the V4V6 region of 16S rRNA. "Others" represents taxa not present at >0.5% in any sample. Numbers of pyrotags per sample are indicated in Table S5. Sample names are the site number followed the core number (e.g. 1374-07 for sample U1374A-7R1).

Figure S5 - Presence of taxa in enrichment experiments from IODP Expedition 330. Media indicated at the top of the chart, each column represents one sample. 1%MB = 1% Marine Broth in artificial seawater; 10%MB = 10% Marine Broth in artificial seawater, HSO = heterotrophic sulfur oxidizer media; HIR = heterotrophic iron reducer media; AIR = autotrophic iron reducer media. Sample names are the site number followed the core number (e.g. 1374-07 for sample U1374A-7R1). Asterisk indicates two enrichment vials were initiated from the same sample.
